# Comprehensive Assessment of Polysaccharides Extracted from Squash by Subcritical Water under Different Conditions

**DOI:** 10.3390/foods13081211

**Published:** 2024-04-16

**Authors:** Yu Zhang, Xun Sun, Bingjie Yang, Fei Li, Guoyong Yu, Jing Zhao, Quanhong Li

**Affiliations:** 1 College of Food Science and Nutritional Engineering, China Agricultural University, Beijing 100083, China; hnzzzhangyu@163.com (Y.Z.); sunxun1212@163.com (X.S.); yangbingjie925@163.com (B.Y.); zhaojing@cau.edu.cn (J.Z.); 2 China National Engineering Research Center for Fruit and Vegetable Processing, Beijing 100083, China; 3 Key Laboratory of Fruit and Vegetable Processing, Ministry of Agriculture, Beijing 100083, China; 4 College of Life Science, Qingdao University, Qingdao 266071, China; pdlifei@163.com; 5 Faculty of Biology, Shenzhen MSU-BIT University, Shenzhen 518172, China; yuguoyong66@163.com

**Keywords:** squash polysaccharides, subcritical water, physiochemical properties, in vitro digestion

## Abstract

The effects of subcritical water microenvironment on the physiochemical properties, antioxidant activity and in vitro digestion of polysaccharides (SWESPs) from squash were investigated. After single-factor experiments, twenty samples were successfully prepared at different extraction temperatures (110, 130, 150, 170 and 190 °C) and extraction times (4, 8, 12 and 16 min). Under a low temperature environment, the whole process was mainly based on the extraction of SWESP. At this time, the color of SWESP was white or light gray and the molecular mass was high. When the temperature was 150 °C, since the extraction and degradation of SWESP reached equilibrium, the maximum extraction rate (18.67%) was reached at 150 °C (12 min). Compared with traditional methods, the yield of squash SWESP extracted by subcritical water was 3–4 times higher and less time consuming. Under high temperature conditions, SWESPs were degraded and their antioxidant capacity and viscosity were reduced. Meanwhile, Maillard and caramelization reactions turned the SWESPs yellow-brown and produced harmful substances. In addition, different SWESPs had different effects on in vitro digestion. In brief, SWESPs prepared under different conditions have different structures and physicochemical properties, allowing the obtainment of the required polysaccharide. Our results show that squash polysaccharides prepared in different subcritical water states had good development potential and application in the food industry.

## 1. Introduction

Squash (*Cucurbita moschata*) is widely distributed worldwide and a rich source of nutrients and active ingredients. Polysaccharides are an important functional active ingredient in squash [1]. Multiple research groups have isolated, purified and determined the chemical structures of squash polysaccharides [2,3]. Meanwhile, the research on squash polysaccharides is mainly to explore their physiological functions and applications in food [4,5]. To the best of our knowledge, squash polysaccharides have a variety of biological activities, including antioxidant, immune regulation, hypoglycemic and anti-inflammatory activities [6,7,8,9,10]. In previous studies, squash polysaccharides have mainly been extracted by hot water, acidic solution, alkali solution, microwave-assisted, ultrasound-assisted and enzyme-assisted techniques [3,11,12,13,14]. However, these extraction methods exhibit certain drawbacks such as their low extraction efficiency and time consuming nature and the solvent residue and environmental pollution produced [15]. Therefore, subcritical water extraction (SWE) has garnered increasing attention owing to its high efficiency, low consumption of resources, environmentally friendly nature, etc., [16].

Subcritical water is water heated above its boiling point (100 °C) but below its critical point (374 °C) while controlling the system pressure within the range 1–22.1 MPa in order to maintain the water in a liquid state [17].

The thermal motion of water molecules intensifies and leads to alterations in their properties under these circumstances [18]. At this moment, the water changes from strong polarity to non-polarity and solutes can be extracted from low to high polarity. The polarity viscosity and surface tension of water decrease as the temperature and pressure increase. Meanwhile, the dielectric constant of water is similar to that of organic solvents and can be controlled by adjusting the temperature [19]. In addition, high temperatures are more likely to weaken hydrogen bonds, which in turn reduces the energy required for fragmentation in solute–matrix interactions, increasing extraction efficiency [20]. No chemicals are used in the entire extraction process; therefore, no waste liquid is generated and the harm to the environment is minimal. Furthermore, diverse polysaccharide structures exhibit distinct biological activities and the structure of polysaccharides can be influenced by the various extraction methods. During polysaccharide extraction, the subcritical water state can lead to breakage of glycosidic bonds, resulting in the formation of smaller molecular mass polysaccharides [21,22]. Intriguingly, this phenomenon may account for the enhanced polysaccharide activity achieved through SWE [23]. Thus, it is crucial to assess the physicochemical characteristics of squash polysaccharides obtained through SWE.

In this study, squash polysaccharides were isolated using subcritical water under different conditions. Furthermore, the yields and physicochemical, rheological, emulsifying and antioxidant properties of the samples were comprehensive evaluated. The rule for SWE in the preparation of squash polysaccharide was expounded. The results are expected to provide reference and commercial applications for the development and utilization of SWESPs.

## 2. Materials and Methods

### 2.1. Materials

Squash (*Guangmi No.* 1) weighing 4.0–5.0 kg was harvested in July 2022 (Guangdong, China). To minimize differences, we selected squash that were the same size and had similar skin and flesh colors for treatment. After the squash was cleaned, the skin and seeds were removed. Next, the flesh of the squash was freeze-dried and crushed (100 mesh). Glucose, galactose, mannose, rhamnose, fucose, xylose, arabinose, glucuronic acid, galacturonic acid, 1,1-diphenyl-2-picrylhydrazyl radical (DPPH) and 2-2′-azino-bis (3-ethylbenzothiazoline-6-sulfonic acid) salt (ABTS) were purchased from Solarbio Technology Co., Ltd. (Beijing, China). α-Amylase (A-3176-500KU, 10 U/mg), pepsin (P6887-5 G, 3706 U/mg), pancreatin (P1750-100 G) and amyloglucosidase (A7420, 31.2 U/mg) were obtained from Sigma-Aldrich Corp. (St. Louis, MO, USA).

### 2.2. Extraction of Squash Polysaccharides

The method used was similar to that of previous work [24], with slight modifications. Briefly, a mixture of 7.5 g squash powder and 300 mL distilled water was introduced into a high-temperature and high-pressure reactor (YZPR-500M, Yanzheng Instrument, Shanghai, China). The reactor was sealed and a high-purity nitrogen gas (99.99%) was employed to maintain the desired pressure. Samples were stirred with an equipped metal stirrer [25]. Thereafter, the stainless-steel reactor was heated via a heating mantle. The extraction process commenced once the temperature reached the designated value. Once the reaction was over, the reactor system was shut down. The reactor was immediately cooled to 40 °C with running tap water. The supernatant was collected through centrifugation (10,000× *g*, 15 min) and mixed with anhydrous ethanol (ethanol final concentration: 90%) at 4 °C for 12 h. Precipitates were obtained, dialyzed (3500 Da, 3 days) and lyophilized. The obtained polysaccharides were named SWESPs (subcritical water extraction squash polysaccharides) and stored at 4 °C for further analysis. The SWESPs had a moisture content of 1.25–1.73%.

First, as the solvent/solid ratio (20, 30, 40, 50, 60 mL/g), extraction time (4, 8, 12, 16, 20 min), pressure (1, 3, 5, 7, 9 MPa) and temperature (110, 130, 150, 170, 190 °C) were the main factors influencing the SWE of the polysaccharides, single-factor experiments were performed on them. The extraction time and extraction temperature had a great influence on the yield. After that, the solvent/solid ratio and extraction pressure were controlled at 40 mL/g and 5 MPa, respectively. The extraction temperature (110, 130, 150, 170, 190 °C) and the extraction time (4, 8, 12, 16 min) were set before beginning the process. The impact of the extraction temperature and duration on the yield of squash polysaccharides was investigated. The yield was calculated as follows:

$$\mathrm{Yield}\;(\%)=\frac{\mathrm{The}\;\mathrm{weight}\;\mathrm{of}\;\mathrm{squash}\;\mathrm{polysaccharide}\;}{\mathrm{The}\;\mathrm{weight}\;\mathrm{of}\;\mathrm{squash}\;\mathrm{powder}}\times 100\%$$

Note: The extraction temperature ranged from 110 to 190 °C and the extraction time ranged from 4 to 16 min, which led to 20 combinations marked by “number1-number2”. For example, “110-4” means the sample was extracted at 110 °C for 4 min.

### 2.3. Chemical Composition of the SWESPs

The neutral sugar content was analyzed using the phenol–sulfuric acid method with D-glucose as the standard [26]. Galacturonic acid (GalA) was tested using the m-hydroxybiphenyl colorimetric procedure [27]. The content of protein was tested with a fully automated Kjeldahl analyzer (K1100, Jinan Hanon Instruments Co., Ltd., Jinan, China) [28]. The total phenolic content was determined via the Folin–Ciocalteu (FC) colorimetric method described by Chaiklahan et al. [29] with gallic acid as the standard.

### 2.4. Monosaccharide Composition of the SWESPs

The method of Li et al. [30] was applied in this part of the experiment. SWESPs (5 mg) were subjected to hydrolysis using trifluoroacetic acid (TFA, 2 mL, 3 M). The hydrolytic solution was transferred to the tube for nitrogen blow-drying. Deionized water (10 mL) was added to the tube and mixed. The mixture was diluted 25 times and centrifuged at 10,000× *g* for 5 min. The sample (5 µL) was evaluated under the following conditions. The mobile phase was H_2_O, NaOH (15 mM) and NaOH (15 mM) + NaOAc (100 mM). The column temperature was 30 °C. Glucose, galactose, mannose, rhamnose, fucose, xylose, arabinose, glucuronic acid and galacturonic acid were used as standards.

### 2.5. Molecular Mass of the SWESPs

The molecular mass of the SWESPs was determined using high-performance gel permeation chromatography (LC-10A, Shimadzu Scientific Instruments, Kyoto, Japan) [31]. The dextran standard (5.0, 11.6, 23.8, 48.6, 80.9, 148.0, 273.0, 409.8 and 667.8 KDa) and SWESP (5 mg/mL) was prepared. The solution was filtered through a 0.22 μm microporous membrane and transferred to the sample vial (1.8 mL). The sample (20 μL) was injected with a flow rate of 0.6 mL/min at 40 °C and the mobile phase was NaCl (0.05 M).

### 2.6. Colorimetric Analysis of the SWESPs

The color parameters (L*, a*, b*) of the SWESPs were measured using a colorimeter (CS-580A, Hangzhou Color Spectrum Technology Co., Ltd., Hangzhou, China) according to the method from previous studies with some modifications [32].

### 2.7. Fourier Transform Infrared (FT-IR) Spectroscopy

The FTIR spectra (NICOLET-6700 spectrometer, Thermo, Waltham, MA, USA) were collected for the SWESPs. The spectrum was recorded at a wavenumber range of 400–4000 cm^−1^ with 16 scans and a resolution of 4 cm^−1^ [33].

### 2.8. Rheological Properties of the SWESPs

The rheological properties of the samples were analyzed using a rheometer (DHR-2, TA Instruments, New Castle, DE, USA) via a method adapted from Li et al. [34]. The viscosities of the SWESP samples (20 mg/mL) were measured by steady-rate shear tests in a shear rate range of 0.01–100 s^−1^ at 25 °C. The angular frequency was 10 rad/s and the strain was 1%. A diameter parallel plate (40 mm) with a gap size of 0.5 mm was used.

### 2.9. Emulsifying Properties of the SWESPs

SWESP solutions (0.5%, *w*/*v*) were prepared by fully dissolving a certain amount of the SWESPs in pure water. Then, SWESP solutions (5 mL) were combined with 5 g of soybean oil. The mixtures were sheared at high speed for 3 min at 12,000 rpm using a high-speed emulsifier (Ultra-Turrax*T 18, IKA, Staufen, Germany) [35].

### 2.10. Antioxidant Activity of SWESP

The DPPH and ABTS radical scavenging capacities were evaluated following the methods described by Zhang et al. [36], with minor modifications. A series of gradient concentrations of polysaccharide solutions (0.3125, 0.625, 1.25, 2.5, 5 mg/mL) were configured and analyzed. DPPH solution (0.1 mM) was composed of DPPH (4.0 mg) and absolute ethanol (100 mL). The polysaccharide solutions were mixed with DPPH solution in an equal volume and reacted at room temperature under dark conditions (30 min). The absorbance was measured at 517 nm. ABTS operating solution was prepared for mixing the ABTS solution (10 mL, 7 mM) with potassium persulfate solution (175 μL, 140 mM) in the dark for 15 h. The same sample solution was mixed with the ABTS operating solution at 37 °C in dark for 6 min. The absorbance was measured at 405 nm. Ascorbic acid served as the positive control for both experiments. The percentage of DPPH and ABTS radical scavenging activities was calculated as follows:

$$\mathrm{DPPH}\;\mathrm{or}\;\mathrm{ABTS}\;\mathrm{radical}\;\mathrm{scavenging}\;\mathrm{activity}\;(\%)=\frac{A_{0}–(A_{s}–A_{c})}{A_{0}}$$

where A_0_ is the absorbance of *DPPH* or *ABTS* solution with distilled water; A_s_ is the absorbance of *DPPH* or *ABTS* solution with sample solution; and A_c_ is the absorbance of sample solution with absolute ethyl alcohol. The IC_50_ is recorded as the sample concentration when the scavenging activity was 50%.

### 2.11. Congo Red Test of the SWESPs

According to the methods of Liang et al. [35], the SWESPs (1 mL, 2.0 mg/mL) were mixed with Congo red (1 mL, 80 μmol/L). NaOH (1 mol/L) was added until the final NaOH concentration reached 0–0.5 mol/L. After the mixture was fully mixed and had been left standing for 10 min, the absorbance was measured by scanning in the range 400–700 nm.

### 2.12. Effect of the SWESPs on the Glycemic Index (GI) of Wheat Flour during In Vitro Digestion

The method for in vitro digestion was the same as hat used by Zhang et al. [28]. The SWESPs (10 mg) and wheat flour (100 mg) were mixed, and the resulting mixture was thoroughly mixed with 10 mL of distilled water. The sample was heated in a 100 °C thermostatic water bath for 25 min before cooling to 37 °C. The gelatinized sample was vacuum freeze-dried and ground. Samples (100 mg) were used to simulate the digestion process of the oral, stomach, and gastrointestinal areas using a simulated digestive system GI20 (NI Ltd., Belfast, UK). During the oral stage (5 min), the sample was blended with 2 mL simulated saliva and added to a rotor to simulate peristaltic digestion. After that, it went through 1.5 h of stomach digestion and 4 h of intestinal digestion. GI values were monitored at different time points (10, 60, 120, 180, 240 and 300 min) using a GM9 glucose analyzer (Analox, Stokesley, UK).

### 2.13. Statistical Analysis

SPSS 20.0 was used to process the data using triplicate measurements for each sample. The figures were printed using Origin 2019b.

## 3. Results and Discussion

### 3.1. Yield of the SWESPs

Yield is a pivotal parameter to evaluate the extraction efficiency during SWESP preparation. Meanwhile, the extraction temperature and time are both crucial parameters in the process of squash polysaccharide extraction. In this work, the effects of solvent/solid ratio, extraction time, extraction pressure and extraction temperature on the yield of squash polysaccharides were studied (Supplementary Figure S1A–D). The results demonstrated that the extraction temperature and time had a great influence on the yield; however, this was not true for the extraction pressure. Therefore, the effects of the extraction temperature (110, 130, 150, 170, 190 °C) and the extraction time (4, 8, 12, 16 min) of the SWE on the yield of polysaccharides from squash were further investigated (Supplementary Figure S1E). Under different extraction conditions, the SWESP yield ranged from 12.43% to 18.67%, indicating that the extraction conditions significantly influenced the yield of SWESPs. Compared with traditional methods (hot water extraction etc.), the yield of squash polysaccharides extracted via SWE is 3–4 times higher and less time consuming [1,3]. Zhang et al. [4] obtained squash polysaccharides via hot water extraction (80 °C, 4 h) and purified them. The yield of squash polysaccharides before and after purification was 5.46% and 1.30%, respectively.

As shown in Supplementary Figure S1E, the most suitable extraction temperature and time for a high yield of SWESPs are 150 °C and 12 min, respectively. The yield of SWESPs initially increased and then decreased with an increase in extraction time at each extraction temperature. This illustrated that the polysaccharides cannot be fully extracted in a short time and will be degraded under the conditions of SWE if the time is too long [36]. In addition, the SWE of SWESPs exhibited a relatively low efficiency at lower temperatures. However, the degradation of SWESPs by subcritical water became more pronounced than the extraction effect when the extraction temperature was excessively high, leading to a significant decreased in the yield of SWESPs. According to the SWESP extraction yield results, extraction temperature had a greater impact on extraction yield than extraction time. When the extraction temperature was lower than 150 °C, the SWESP extraction efficiency was higher than the degradation efficiency; therefore, the extraction rate increased as the temperature increased. At about 150 °C, the extraction and degradation of the SWESPs reached a relative balance; hence, extraction time at this time had no significant effect on the extraction rate. When the temperature was further increased, the degradation rate of the SWESPs exceeded the extraction rate, resulting in a decrease in the extraction rate of the SWESPs. Meanwhile, some undesirable substances were also produced at high temperatures, such as furfurals and the products of Maillard reactions [36].

### 3.2. Chemical Composition of the SWESPs

**Figure 1:**
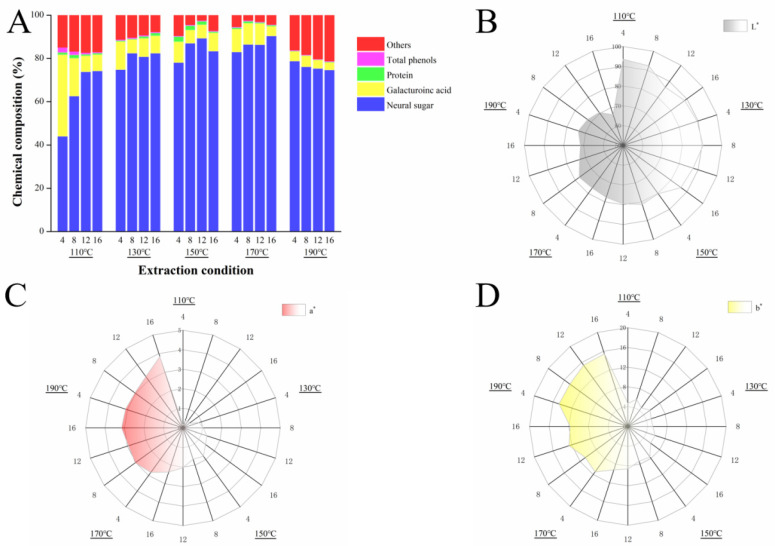
The chemical composition (**A**) and colorimeter analysis (**B**–**D**) of SWESPs. The solvent/solid ratio and extraction pressure were 40 mL/g and 5 MPa, respectively.

The chemical composition of the SWESPs under different extraction conditions is shown in in Figure 1A. The contents of neutral sugar and GalA were 44.10–90.42% and 3.48–37.72%, respectively. Neutral sugar is the main component of squash polysaccharides and are derived from the extraction or degradation of the main or side chains of polysaccharides and lignocellulose (cellulose or hemicellulose) [37]. As the extraction temperature increased, the content of neutral sugars in the SWESPs gradually increased. Notably, when the extraction temperature was 190 °C, the content of neutral sugars decreased, which may be the result of partial neutral sugar reactions or the degradation of the SWESPs. Interestingly, the change in GalA content was the opposite to that of the neutral sugar content. The GalA content was relatively high and without excessive degradation when the extraction temperature was lower. However, the GalA content exhibited a significant decline under high temperature conditions, which may be attributed to the accelerated thermal degradation of the SWESPs by the high temperature and prolonged extraction [38]. In addition, the increase in temperature will also lead to an increased in the ionization constant of water, thereby enhancing the acidity of the solution and further enhancing the acidolysis of pectin polysaccharides [39]. At an extraction temperature of 110 °C, the prolongation of extraction time also contributed to the sharp decrease in GalA content.

As can be seen, each sample contained a small amount of protein and total phenol. With an increased in extraction temperature and extraction time, the total phenol content in the system decreased due to the unstable structure. In addition, the unknown parts of the SWESPs may consist of ash, pigments, etc. Simultaneously, at 190 °C, the proportion of unknown components increased significantly, which may have been caused by the reaction of the components in the system to produce new substances under the high-temperature and high-pressure environment.

### 3.3. Monosaccharide Composition of the SWESPs

Based on the high-performance ion chromatography (HPIC), the SWESPs were mainly composed of glucose (Glu, 62.58–101.22 mg/g), galactose (Gal, 9.27–16.31 mg/g), rhamnose (Rha, 0.00–1.59 mg/g), arabinose (Ara, 0.10–3.08 mg/g) and galacturonic acid (GalA, 2.97–55.28 mg/g) (Table 1), which determined the physicochemical and structural properties of the SWESPs. Glucose and galacturonic acid were the main monosaccharide components of the SWESPs, followed by arabinose, galactose and rhamnose. This result was consistent with Section 3.2 and with previous studies regarding the types of squash monosaccharides, but the quantities were slightly different [1,30].

For all SWESP samples, it was observed that the contents of Glu and Gal increased significantly with higher extraction temperatures and longer extraction times, particularly when the temperature exceeded 150 °C. Subcritical water degraded more cellulose and hemicellulose in squash pulp at relatively high temperature and pressure, resulting in an increase in the content of Glu. The increase in Gal may be due to the breakage of SWESP side chains, leading to the formation of new polysaccharides. This is consistent with the aforementioned extraction and degradation of polysaccharides using subcritical water. Inversely, the content of Rha, Ara and GalA in the SWESP samples declined with an increase in extraction temperature. Since GalA and Rha were the main components of homogalacturonic acid (HG) and rhamnogalacturonic acid (RG) frameworks, it was inferred that their frameworks were destroyed under high-temperature and high-pressure conditions in subcritical water [36,40,41]. Intriguingly, Rha was not detected in 190-12 and 190-16. Moreover, the ratio of the sum of Ara and Gal to Rha can be used to analyze the structure and side chain ingredients of pectin polysaccharides [40,42]. The increase in temperature led to a significant rise in the ratio, indicating an augmented presence of hairy regions and side chains in the SWESP samples. Hence, it could play a guiding role in the monosaccharide composition, monosaccharide content and molecular mass of extracted polysaccharides.

### 3.4. Molecular Mass of the SWESPs

The standard curve was derived from different molecular mass dextrans (5.0, 11.6, 23.8, 48.6, 80.9, 148.0, 273.0, 409.8, 667.8 KDa). According to the standard curve equation (log M_w_ = −0.1939X + 12.27, R^2^ = 0.9924, where M_w_ is the molecular mass and X is the retention time), the molecular masses of the SWESPs were calculated and are shown in Table 1. The M_w_s of the SWESPs were in the range 30.826–478.411 KDa, indicating a relatively broad range and a higher molecular mass at low temperature. With an increased in temperature and time, the molecular mass of the SWESPs showed a significant decreasing trend. This result was mainly attributable to two aspects: (1) the state of high temperature and high pressure led to the breaking of the glycosidic bonds of macromolecular polysaccharides, forming more low-molecular-mass polysaccharides [43] and (2) the increased in extraction time prolonged the time the SWESPs were in a serious environment, which in turn led to the degradation of the polysaccharides [41]. When the temperature was higher than 150 °C, the degradation rate of the polysaccharides increased significantly, indicating that temperature had a great influence on the molecular mass of polysaccharides in water in the subcritical state. It is noticeable that the ideal molecular mass polysaccharide can be obtained by changing the temperature of the subcritical water and the extraction time during the extraction process. Polysaccharides with different molecular masses have different roles in food processing. For example, polysaccharides with molecular masses of 600–700 kDa were more suitable as gelling or thickening agents and high-molecular-mass polysaccharides were more suitable for making food preservation films; however, low-molecular-mass polysaccharides may be more biologically active [1,44]. Therefore, a series of polysaccharides with different molecular masses prepared in different subcritical water states have good development potential in the food industry field. Through the analysis of extraction conditions and the properties of the SWESPs, the industrial production and application of SWESPs can be further realized.

### 3.5. Colorimetric Analysis of the SWESPs

Color is one of the most important organoleptic properties of polysaccharides and is affected by components such as pigments. Meanwhile, some physical and chemical reactions can also lead to color changes in polysaccharides, such as Maillard reactions, caramelization and so on [45]. The principle of color analysis is to apply the Hunter Lab system, where L* represents brightness, a* represents red (+) and green (−) chromaticity and b* represents yellow (+) and blue (−) chromaticity [46].

As illustrated in Figure 1B–D, the ranges of the L*, a* and b* values were 66.39–93.58, 0.16–3.84 and 4.64–15.46, respectively. As the extraction temperature increased, the L* value gradually decreased. In contrast, the a* and b* values gradually increased. In low-temperature areas, the color of the SWESPs was snow white. With an increase in extraction temperature and time, the SWESPs first changed from white to dark gray. The results from this investigation were inconsistent with the findings from the previous investigation performed by Zhang et al. [36], which may be attributed to the structure of the polysaccharides, the raw material pigments and the composition of ingredients. When the temperature was higher than 170 °C, the SWESPs turned yellow-brown, which was consistent with the experimental results. In addition, in the low-temperature region, the color change range of the SWESPs was small, while at high temperatures, the color of the SWESPs changed sharply. That is to say, high temperatures had a great effect on the color of the SWESPs and was also a major factor in the formation of red and yellow colors. The values of a* and b* showed the same trend as in Figure 1, and the ratio of b* to a* was always greater than 1, which meant that yellow was more dominant than red in the color change process [47]. After the polysaccharides underwent degradation and a series of reactions at high extraction temperatures, new substances such as furfurals and 5-hydroxymethylfurfural appeared, resulting in the color of the SWESPs changing from white to yellow-brown [36]. Moreover, Maillard and caramelization reactions also affected the color of the SWESPs [32,48]. The specific change mechanism of the polysaccharide color change needs to be further studied. In addition to this, the influence of polysaccharide color in the food industry should also be considered, so that the corresponding extraction conditions can be controlled to meet any requirements when preparing polysaccharides with SWE.

### 3.6. Fourier-Transform Infrared (FT-IR) Spectroscopy

**Figure 2:**
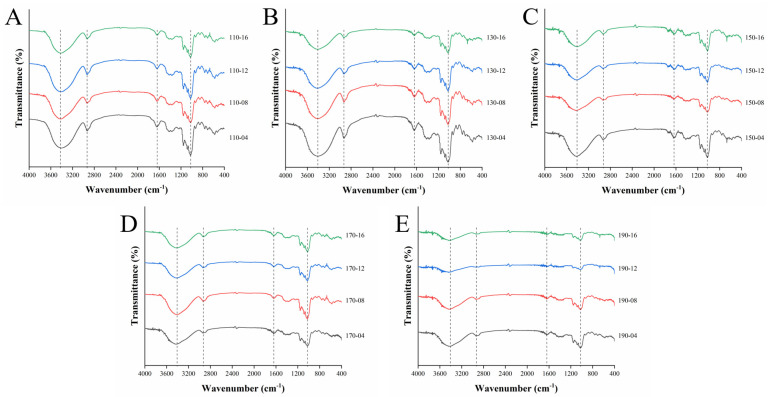
FT−IR spectrum analysis of SWESPs. (**A**) Extraction at 110 °C for different times (4, 8, 12, 16 min). (**B**) Extraction at 130 °C for different times (4, 8, 12, 16 min). (**C**) Extraction at 150 °C for different times (4, 8, 12, 16 min). (**D**) Extraction at 170 °C for different times (4, 8, 12, 16 min). (**E**) Extraction at 190 °C for different times (4, 8, 12, 16 min). The solvent/solid ratio and extraction pressure were 40 mL/g and 5 MPa, respectively.

The changes in the functional groups of all the samples were studied via FT-IR spectroscopy. A high similarity in FT-IR absorption patterns was found in all SWESP samples (Figure 2). The broad band between 3000 and 3700 cm^−1^ corresponds to the O-H bond stretching vibration [49]. Bands near 2940 cm^−1^ are associated with the stretching vibration of the C-H bond [50]. The weak absorption band at the 2330 and 2370 cm^−1^ regions indicates some amidated components with N-H stretching. Additionally, the peak occurring at 1640 cm^−1^ correspond to the asymmetric stretching vibrations of the carboxyl group, confirming the presence of uronic acid in the SWESPs [51]. The signals in the ranges 800–1200 cm^−1^ and 1000–1200 cm^−1^ result from the fingerprint regions of pectic polysaccharides and the sugar ring backbones of C-O and C-C, respectively. The absorption peak at 850 cm^−1^ indicated the existence of α-configuration pyranose, and the absorption vibrations at 2940, 1370 and 1235 cm^−1^ were the main peaks assigned to the asymmetric and symmetric stretching vibration of the CH_2_ of the pyranose ring for the SWESP samples [52,53]. The peaks occurring at 1080, 1030 and 928 cm^−1^ corresponded to Gal and Glu [25,54]. The strength of these characteristic peaks was also related to the change in their contents during the extraction process. It is worth noting that the infrared spectral transmittance and the peak size under different extraction conditions were different. This might be explained by research showing that the extraction, degradation and interaction of polysaccharides in different SWE states.

### 3.7. Rheological and Emulsifying Properties of the SWESPs

**Figure 3:**
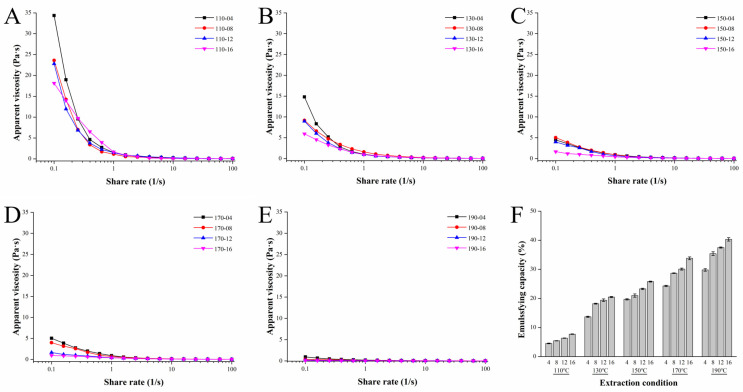
Analysis of the rheological (**A**–**E**) and emulsifying properties (**F**) of SWESPs. (**A**) Extraction at 110 °C for different times (4, 8, 12, 16 min). (**B**) Extraction at 130 °C for different times (4, 8, 12, 16 min). (**C**) Extraction at 150 °C for different times (4, 8, 12, 16 min). (**D**) Extraction at 170 °C for different times (4, 8, 12, 16 min). (**E**) Extraction at 190 °C for different times (4, 8, 12, 16 min). The solvent/solid ratio and extraction pressure were 40 mL/g and 5 MPa, respectively.

The apparent viscosities of the SWESP solutions were assessed using steady-state flow measurements. As can be seen from Figure 3A–E, the flow curves trended similarly for all SWESP samples; they decreased with increasing shear rate, dropping sharply and then flattening out. Interestingly, the apparent viscosity of the SWESPs was decreased with increasing extraction temperature and time. All solutions exhibited pronounced shear-thinning pseudoplastic behavior. It is known that the rheological behavior of SWESPs is closely related to monosaccharide composition, molecular mass and GalA content [55]. Hence, the results were consistent with the findings from the aforementioned investigation. High temperatures led to a decrease in the molecular weight of the SWESPs and thus a decrease in the apparent viscosity. Moreover, the long extraction process made the flow curve relatively flat. These results demonstrate that the apparent viscosity of the SWESPs was significantly influenced by both the extraction temperature and duration. The different rheological properties of the SWESPs affected their biological functions, processing and transport modes [23,36]. Therefore, predicting and explaining the rheological properties of the SWESPs plays an important role in food processing, manufacturing and development. According to the results of this research, different extraction conditions can be utilized to prepare SWESPs with desirable rheological properties.

The emulsifying properties of all 20 SWESP samples are shown in Figure 3F. The molecular mass, composition of monosaccharides, content of hydrophobic functional groups and the degree of branching of the SWESPs are all key factors that determine their emulsifying capacity [56]. The emulsifying capacity of the SWESPs ranges from 4.55 to 40.30% and increased with an increase in extraction temperature and extraction time; however, it is more affected by temperature. According to the results in Section 3.4, under high extraction temperatures, the SWESPs were degraded easily, resulting in SWESP sample with low molecular mass, thus improving the emulsifying capacity. In addition, GalA content and a small amount of protein were also factors that affected the emulsifying capacity of the SWESPs [57]. The ideal polysaccharide emulsifying capacity can also be controlled by controlling the SWE extraction conditions, which provides a theoretical basis for precise polysaccharide preparation and industrial application.

### 3.8. Antioxidant Activity of SWESP

**Figure 4:**
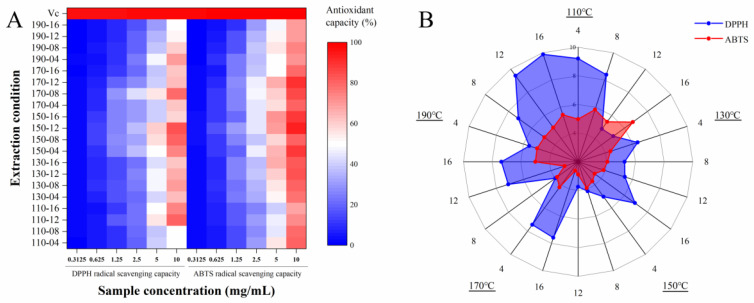
DPPH and ABTS radical scavenging activities (**A**) and in vitro IC_50_ value (**B**) of SWESPs. The extraction temperature ranged from 110 to 190 °C and the extraction time ranged from 4 to 16 min, which led to 20 combinations marked by “number1–number2”. The solvent/solid ratio and extraction pressure were 40 mL/g and 5 MPa, respectively.

In this study, the antioxidant activity (DPPH and ABTS radical scavenging capacities) of SWESPs and a positive control (Vc) were investigated. The results are shown in Figure 4. DPPH is a stable nitrogen-centered free radical. In the presence of free radical scavengers, the single electron of DPPH is captured and the color becomes lighter. The process is widely used in the determination of the antioxidant capacity of natural products in vitro due to its simple operation and good repeatability. As can be seen from Figure 4A, in the range 0.3125–10 mg/mL, the antioxidant activities of all SWESP samples showed an obvious concentration dependence. In addition, it was evident that the effect of the extraction temperature and time on the free radical scavenging ability of DPPH played an important role. The DPPH radical scavenging capacity of the SWESPs was 0.65–60.38%. The samples obtained at 150-08 and 150-12 showed the best free radical scavenging ability during the assay. At low temperatures, prolonging the extraction time can improve the yield of SWESPs and effectively improve the antioxidant capacity. The opposite result was obtained at high temperatures, a result which may be related to the degradation and reaction of SWESPs [58]. Moreover, as illustrated in Figure 4B, the IC_50_ values of the SWESP samples showed a similar trend to the free radical scavenging activity. The samples of 150-10, 150-15 and 170-08 had relatively low IC_50_ values. The results from the investigation were inconsistent with the findings from the previous investigation performed by Zhang et al. [36]. This result may be related to the type of polysaccharides, monosaccharide composition and molecular mass, among other reasons [59]. Since the antioxidant capacities of the SWESP samples were numerous and complex, further research was needed, especially regarding the degradation and interaction of polysaccharides. The ABTS radical cation is formed by the oxidation of ABTS with potassium persulfate. The color of the stock solution is blue-green, and after adding the polysaccharide solution, the antioxidant components contained in the substance react with the ABTS radical cation, causing the reaction system to fade. This is a model for evaluating the antioxidant activity of polysaccharides. It can be seen from Figure 4A that within the measurement range (0.3125–10 mg/mL), the scavenging ability of ABTS gradually enhanced with an increased SWESP concentration, which was consistent with the results of the DPPH radical scavenging capacity test. The data distribution of the ABTS radical scavenging capacity of SWESPs at different extraction temperatures and extraction times was also similar to the previous results. The ABTS radical scavenging capacity of the SWESPs was 0.25–93.00%. The difference was that the scavenging capacity of the SWESPs for ABTS was stronger than that for DPPH. Therefore, the red area was smaller than the blue area (Figure 4B). Furthermore, compared with V_C_, SWESPs had slightly weaker scavenging capacity for DPPH and ABTS radicals. Compared with the traditional hot water extraction method, the DPPH and ABTS radical scavenging capacity of SWESPs extracted via the subcritical water method was significantly improved [60].

Polysaccharide is a natural biological macromolecule, and its activity is closely related to its structure, composition, interaction and state, among other things [61]. However, the study of a single polysaccharide is not enough to clarify the relationship between its structural composition, physicochemical properties and biological activity. Therefore, a series of polysaccharides were prepared and analyzed from the same raw material using different extraction states, which can help to distinguish the differences in the physicochemical properties and bioactivities of polysaccharides. It is hoped that the relevant properties and activities of SWESPs can be elucidated from the extraction conditions of different temperatures and times.

### 3.9. Triple Helix of SWESP

**Figure 5:**
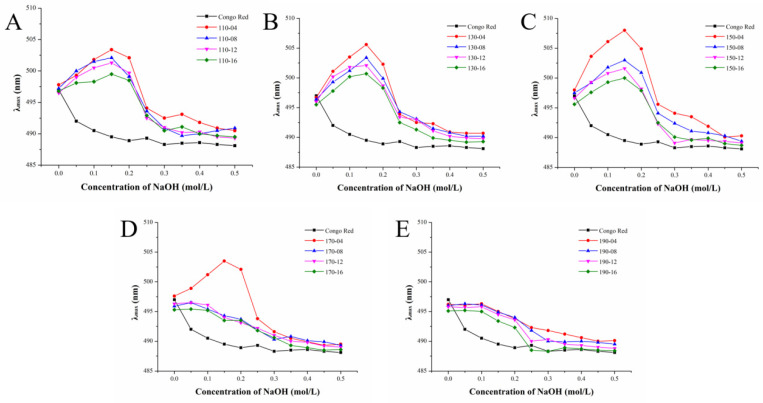
Maximum absorption λ_max_ of SWESP–Congo red complexes at different concentrations of NaOH. (**A**) Extraction at 110 °C for different times (4, 8, 12, 16 min). (**B**) Extraction at 130℃ for different times (4, 8, 12, 16 min). (**C**) Extraction at 150 °C for different times (4, 8, 12, 16 min). (**D**) Extraction at 170 °C for different times (4, 8, 12, 16 min). (**E**) Extraction at 190 °C for different times (4, 8, 12, 16 min). The solvent/solid ratio and extraction pressure were 40 mL/g and 5 MPa, respectively.

Congo red can combine with some polysaccharides to form a complex, resulting in an increase in their maximum absorption wavelength (λ_max_). The maximum absorption wavelength of the complex is red shifted compared to Congo red. Within a certain concentration range, the characteristic change manifested as the maximum absorption wavelength becomes purplish red. Then, the NaOH degree is greater than a certain value, the intermolecular hydrogen bond breaks down, the spiral structure dissolves and the complex cannot be formed. The maximum absorption wavelength drops sharply. The results are illustrated in Figure 5. Except for those extracted at 190 °C, all polysaccharides had triple helix structures, suggesting that a high-temperature environment could damage the triple helix structure of polysaccharides. Under 170 °C, only polysaccharides extracted for 4 min contained trihelix structure, which proved that the longer extraction time, the more easily the trihelix structure of polysaccharides was destroyed. In addition, with the increase in temperature, the maximum wavelength increased first and then decreased, while with the increase in extraction time, the maximum wavelength decreased gradually. The maximum wavelength increases at low concentrations of NaOH, while high concentrations of NaOH can make the conformational structure of the polysaccharide gradually become unstable and even fracture, which may be caused by severe β-elimination fracture [62]. What is more, the regular helical structure of pectin in alkaline solution is related to its special regional structure [63]. Therefore, SWESPs with good triple helix structures can be obtained by extracting at relatively low temperatures and with short extraction times.

### 3.10. In Vitro Digestibility of Gelatinized Wheat Flour Mixed with SWESPs

**Figure 6:**
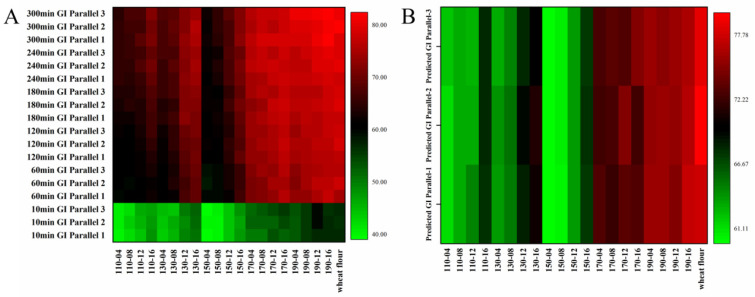
The GI at different times (**A**) and the predicted GI (**B**) of gelatinized wheat flour and gelatinized wheat flour/SWESP mixtures. The extraction temperature ranged from 110 to 190 °C and the extraction time ranged from 4 to 16 min, which led to 20 combinations marked by “number1–number2”. The solvent/solid ratio and extraction pressure were 40 mL/g and 5 MPa, respectively.

It can be seen from Figure 6A that from 10 to 300 min of digestion, the GI value increases gradually, especially for 10–60 min. The GI value of wheat flour increased from 56.64 to 82.03. After the addition of SWESPs, the GI values of all samples decreased to different degrees, except for 190-12 and 190-16. At extraction temperatures of 110–150 °C, SWESPs were able to significantly reduce GI values, especially for 110-04, 150-04 and 150-08 samples. At an extraction temperature of 170 °C, SWESPs had only a weak effect on lowering GI values. In addition, the higher the extraction temperature and the longer the extraction time, the weaker the effect of polysaccharides in reducing the GI value. Figure 6B shows the predicted GI values of 78.62 and 60.03–76.84 for gelatinized wheat flour and wheat flour/SWESP mixtures, respectively. Their trends were similar to the trend of GI value changes throughout the in vitro simulated digestion. The addition of SWESPs can protect the starch from being partially broken during the pasting process. The unbroken starch was difficult to digest, resulting in a low rapidly digestible starch content [64,65]. In addition, there was an electrostatic interaction between SWESPs and starch granules and the SWESPs were surrounded by the surface of the starch granule via hydrogen bonding between the SWESPs and the leached starch. The process hindered the enzymatic hydrolysis of starch and promoted the increase in slowly digestible starch and resistant starch content [66]. Moreover, these inhibitory effects were enhanced with increasing pectin content and molecular weight [67]. The triple helix structure of SWESPs may also be responsible for the lower GI values.

## 4. Conclusions

SWE is an environmentally friendly technique that has been demonstrated to be an excellent method for squash polysaccharide extraction. In the present study, single-factor experiments were first conducted. The results showed that the extraction temperature and time had a great influence on the yield. After that, the properties of a total of 20 samples were investigated at different extraction temperatures (110, 130, 150, 170 and 190 °C) and extraction times (4, 8, 12 and 16 min). The results showed that the extraction temperature played a more important role than the extraction time. Changing the extraction temperature and time has a great influence on the extraction rate, molecular mass, monosaccharide composition, color, viscosity properties, emulsifying properties, antioxidant capacity, triple helix structure and in vitro digestibility of SWESPs. Therefore, the correlation between them was comprehensively evaluated and a database of SWESPs prepared from subcritical water was initially established. According to the needs of the food industry, different extraction methods are adopted to produce the corresponding SWESPs. Specifically, at low temperatures, due to the high molecular mass and viscosity characteristics, SWESPs can be used as gels or thickeners. The SWESPs obtained at about 150 °C have good potential as a kind of antioxidant and can be obtained in large quantities. High-temperature-extracted SWESPs were suitable for related food processing applications due to their unique color, low molecular weight and low viscosity. The triple helix structure of SWESPs occurred at low temperatures and with short extraction times. In addition, the 110-04, 150-04 and 150-08 SWESP samples had a good ability to lower GI values. Meanwhile, the pectin content, molecular weight and triple helix structure of SWESPs significantly affected the GI value. Hence, according to the law that SWESPs prepared under different conditions have different structures and physicochemical properties and actual demand, SWESPs can be extracted for use as gels, thickeners and antioxidant supplements. In summary, the SWE method is expected to important for obtaining squash polysaccharides that can be used in the food industry.

## Figures and Tables

**Table 1:** The monosaccharide composition and molecular mass of SWESPs.

SWESP		M_w_ (10^4^ Da)	Monosaccharide Composition (mg/g)				
Temperature (°C)	Time (min)		Glucose	Galactose	Rhamnose	Arabinose	Galacturonic Acid
110	4	47.84 ± 3.56 ^a^	65.32 ± 0.44 ^i^	10.38 ± 0.56 ^hi^	1.34 ± 0.04 ^c^	3.08 ± 0.17 ^a^	55.28 ± 0.10 ^a^
	8	39.77 ± 3.02 ^b^	66.43 ± 0.08 ^i^	9.64 ± 0.33 ^ij^	1.27 ± 0.01 ^d^	2.95 ± 0.14 ^b^	53.71 ± 0.08 ^bc^
	12	34.58 ± 2.58 ^d^	62.58 ± 0.71 ^j^	10.56 ± 0.45 ^ghi^	1.45 ± 0.02 ^b^	3.01 ± 0.05 ^ab^	54.09 ± 1.21 ^b^
	16	25.93 ± 1.34 ^g^	63.47 ± 0.56 ^j^	10.03 ± 0.71 ^ij^	1.03 ± 0.06 ^fg^	2.82 ± 0.08 ^c^	52.98 ± 0.87 ^c^
130	4	38.90 ± 0.93 ^c^	69.51 ± 1.01 ^gh^	9.27 ± 0.16 ^j^	1.59 ± 0.09 ^a^	2.54 ± 0.06 ^de^	50.25 ± 0.55 ^e^
	8	29.33 ± 2.01^e^	70.44 ± 0.92 ^g^	11.54 ± 0.51 ^efg^	1.24 ± 0.04 ^d^	2.60 ± 0.07 ^d^	53.09 ± 0.37 ^c^
	12	27.47 ± 1.26 ^f^	68.39 ± 1.45 ^h^	10.39 ± 0.28 ^hi^	1.35 ± 0.04 ^c^	2.47 ± 0.10 ^e^	51.38 ± 0.29 ^d^
	16	10.24 ± 1.35 ^j^	65.27 ± 0.88 ^i^	11.92 ± 0.09 ^ef^	1.17 ± 0.05 ^e^	2.03 ± 0.04 ^f^	47.05 ± 0.14 ^f^
150	4	12.77 ± 0.61 ^h^	86.32 ± 1.21 ^e^	12.37 ± 0.34 ^de^	1.08 ± 0.04 ^f^	1.96 ± 0.06 ^f^	37.92 ± 0.43 ^h^
	8	9.86 ± 0.93 ^k^	85.49 ± 0.63 ^e^	10.55 ± 0.25 ^ghi^	0.95 ± 0.08 ^h^	2.08 ± 0.05 ^f^	40.51 ± 0.79 ^g^
	12	6.24 ± 1.08 ^m^	79.27 ± 0.79 ^f^	11.22 ± 0.73 ^fgh^	0.99 ± 0.03 ^gh^	1.74 ± 0.04 ^g^	38.47 ± 0.58 ^h^
	16	4.80 ± 0.54 ^n^	80.35 ± 0.32 ^f^	12.89 ± 0.12 ^d^	0.73 ± 0.04 ^i^	1.59 ± 0.09 ^h^	35.00 ± 0.94 ^i^
170	4	10.85 ± 2.10 ^i^	91.27 ± 0.77 ^c^	14.88 ± 0.36 ^c^	0.51 ± 0.00 ^j^	0.87 ± 0.02 ^i^	12.85 ± 0.15 ^k^
	8	6.34 ± 1.21 ^m^	90.35 ± 1.00 ^cd^	15.41 ± 0.90 ^abc^	0.37 ± 0.01 ^k^	0.92 ± 0.02 ^i^	16.27 ± 0.33 ^j^
	12	4.80 ± 0.45 ^n^	91.01 ± 1.31 ^c^	16.01 ± 0.77 ^ab^	0.26 ± 0.02 ^lm^	0.89 ± 0.05 ^i^	13.58 ± 0.27 ^k^
	16	3.91 ± 0.84 ^p^	89.20 ± 1.04 ^d^	15.20 ± 0.35 ^bc^	0.30 ± 0.01 ^l^	0.67 ± 0.01 ^j^	10.03 ± 0.19 ^m^
190	4	7.53 ± 0.92 ^l^	101.22 ± 0.49 ^a^	16.31 ± 0.94 ^a^	0.21 ± 0.01 ^mn^	0.44 ± 0.03 ^k^	11.38 ± 0.58 ^l^
	8	4.53 ± 0.37 ^o^	100.37 ± 0.56 ^ab^	15.29 ± 0.58 ^bc^	0.15 ± 0.02 ^n^	0.53 ± 0.04 ^k^	7.56 ± 0.24 ^n^
	12	3.92 ± 0.44 ^p^	99.25 ± 0.20 ^b^	13.05 ± 0.64 ^d^	-	0.29 ± 0.01 ^l^	4.29 ± 0.17 ^o^
	16	3.08 ± 0.28 ^q^	100.02 ± 0.38 ^ab^	15.86 ± 0.83 ^abc^	-	0.10 ± 0.00 ^m^	2.97 ± 0.09 ^p^

Values in the same column with different letters are significantly different at *p* < 0.05.

## Data Availability

The original contributions presented in the study are included in the article and Supplementary Materials, further inquiries can be directed to the corresponding author.

## Supplementary Materials

The following supporting information can be downloaded at: https://www.mdpi.com/article/10.3390/foods13081211/s1, Figure S1: Influence of different extraction factors on polysaccharide yield (A) solvent/solid ratio; (B) extraction time; (C) extraction pressure; and (D) extraction temperature. The polysaccharide yield (E) and chemical composition (B) of SWESP.
