# Supplementary material for: Comprehensive Assessment of Polysaccharides Extracted from Squash by Subcritical Water under Different Conditions
Source: Foods. 2024 Apr 16;13(8):1211. doi: 10.3390/foods13081211 (PMC11049192; doi:10.3390/foods13081211)
Supplement: Supplementary file 1 [file foods-13-01211-s001.zip › foods-2936888-Supplementary materials-4.19.pdf]

Supplementary data:

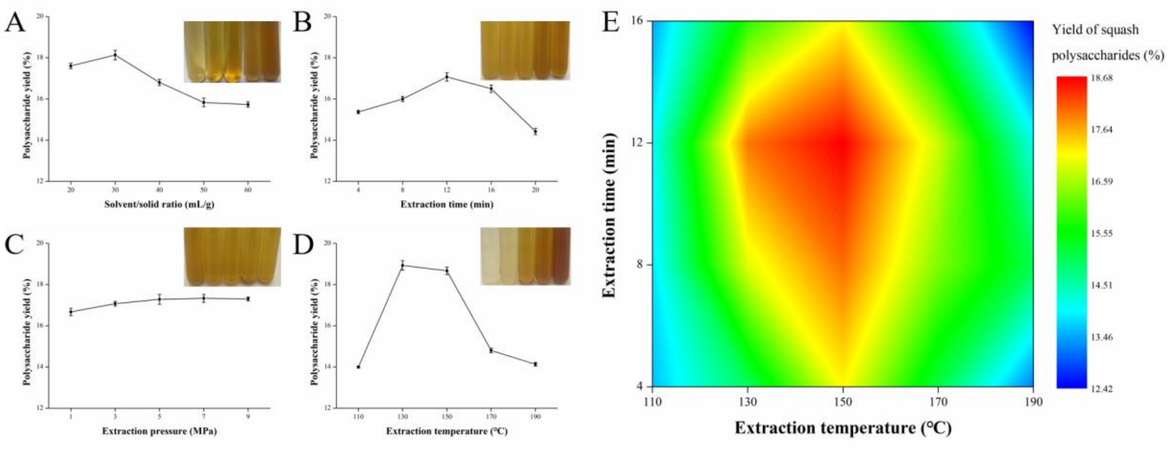

Supplementary Figure S1 Influence of different extraction factors on polysaccharide yield (A) solvent/solid ratio; (B) extraction time; (C) extraction pressure; and (D) extraction temperature. (E) Effects of different extraction temperature and extraction time on SWESP yield (fixed extraction pressure and solvent/solid ratio).
